# Towards Sustainable Production of Biofuels from Microalgae

**DOI:** 10.3390/ijms9071188

**Published:** 2008-07-09

**Authors:** Vishwanath Patil, Khanh-Quang Tran, Hans Ragnar Giselrød

**Affiliations:** 1 Department of Plant and Environmental Sciences, The Norwegian University of Life Sciences, P.O. Box 5003, N-1432 Ås, Norway; 2 Department of Mathematical Sciences and Technology, The Norwegian University of Life Sciences, P.O. Box 5003, N-1432 Ås, Norway

**Keywords:** Biomass, Biofuel, Biodiesel, Hydrothermal liquefaction, microalgae, Pyrolysis, Sustainable energy

## Abstract

Renewable and carbon neutral biofuels are necessary for environmental and economic sustainability. The viability of the first generation biofuels production is however questionable because of the conflict with food supply. Microalgal biofuels are a viable alternative. The oil productivity of many microalgae exceeds the best producing oil crops. This paper aims to analyze and promote integration approaches for sustainable microalgal biofuel production to meet the energy and environmental needs of the society. The emphasis is on hydrothermal liquefaction technology for direct conversion of algal biomass to liquid fuel.

## 1. Introduction

The global economy literally runs on energy. An economic growth combined with a rising population has led to a steady increase in the global energy demands. If the governments around the world stick to current policies, the world will need almost 60% more energy in 2030 than today, of this 45% will be accounted by China and India together [1]. Transportation is one of the fastest growing sectors using 27% of the primary energy [2]. The continued use of fossil fuels is not sustainable, as they are finite resources [3], and their combustion will lead to increased energy-related emissions of green house gases (GHG) viz., carbon dioxide (CO_2_), sulfur dioxide (SO_2_) and nitrogen oxides (NO*_x_*). The future reductions in the ecological footprint of energy generation will reside in a multi-faceted approach that includes nuclear, solar, hydrogen, wind, and fossil fuels (from which carbon is sequestered), and biofuels [4–6]. Biofuel can be broadly defined as solid, liquid, or gas fuel consisting of, or derived from biomass. Rudolph Diesel first demonstrated the use of biodiesel from a variety of crops in 1900. However, the widespread availability of inexpensive petroleum during the 20^th^ century determined otherwise. Generally, shifting society'sdependence away from petroleum to renewable biomass contributes to the development of sustainable industrial society and effective management of GHG [7, 8]. A major criticism often leveled against biomass, particularly against large-scale fuel production, is that it will consume vast swaths of farmland and native habitats, drive up food prices, and result in little reduction in GHG emissions [5, 9–10]. However, this so-called “food versus fuel” controversy appears to have been exaggerated in many cases. Credible studies show that with plausible technology developments, biofuels could supply some 30% of global demand in an environmentally responsible manner without affecting food production [11].

At the moment, only biodiesel and bioethanol are produced on an industrial scale. They are the petroleum replacement for internal combustion engines, and are derived from food crops such as sugarcane, sugar beet, maize (corn), sorghum and wheat, although other forms of biomass can be used, and may be preferable [12]. The most significant concern is the inefficiency and sustainability of these first generation biofuels. In contrast, the second generation biofuels are derived from non-food feedstock. They are extracted from microalgae and other microbial sources, ligno-cellulosic biomass, rice straw and bio-ethers, and are a better option for addressing the food and energy security and environmental concerns. Microalgae, use a photosynthetic process similar to higher plants and can complete an entire growing cycle every few days. In fact, the biomass doubling time for microalgae during exponential growth can be as short as 3.5h [13]. Some microalgae grow heterotrophically on organic carbon source. However, heterotrophic production is not efficient as using photosynthetic microalgae [13], because the renewable organic carbon source required is ultimately produced by photosynthetic crop plants.

Microalgae are veritable miniature biochemical factories, and appear more photosynthetically efficient than terrestrial plants [14] and are efficient CO_2_ fixers [15]. The ability of algae to fix CO_2_ has been proposed as a method of removing CO_2_ from flue gases from power plants, and thus can be used to reduce emission of GHG. Many algae are exceedingly rich in oil, which can be converted to biodiesel. The oil content of some microalgae exceeds 80% of dry weight of algae biomass [13, 16]. The net annual harvest of algal biomass cultivated in subtropical areas can be as high as 40 tons ha^−1^ (dry matter), even higher if CO_2_ is supplied [17]. It is possible to produce about 100 g m^−2^ d^−1^ of algal dry matter in simple cultivation systems [18]. In theory, high oil content algae could produce almost 100 times of soybean per unit area of land [19]. The calculations made by Chisti [20] clearly demonstrate the strong scenario for microalgal biofuels. The use of algae as energy crops has potential, due to their easy adaptability to growth conditions, the possibility of growing either in fresh-or marine waters and avoiding the use of land. Furthermore, two thirds of earth'ssurface is covered with water, thus algae would truly be renewable option of great potential for global energy needs. This paper aims to analyze and promote integration approaches for sustainable microalgal biodiesel production, with emphasis on hydrothermal technology for direct liquefaction of algal biomass with no need to dry the feedstock.

## 2. Integrated Biodiesel Production for Microalgae

**Figure 1.**
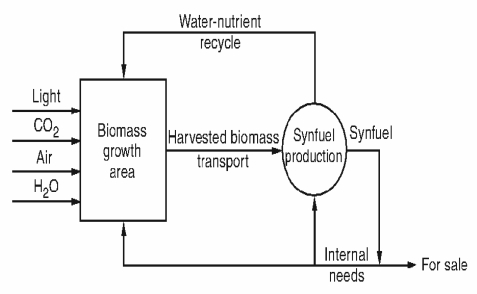
A conceptual model for integrated biomass production and conversion integration system.

The key for large scale production of biofuels is to grow suitable biomass species in an integrated biomass production conversion system (IBPCS) at costs that enable the overall system to be operated at a profit. The illustration in Figure 1 is a conceptual model for integrated biomass production [17] that can be adopted for microalgal biodiesel production. The design of an IBPCS requires the combination and optimization of several factors such as biomass culture, growth management, transport to conversion plants, drying, product separation, recycling, waste (liquid and solid) management, transport of saleable products and marketing. These factors can be simplified and reduced to three main groups; culturing of microalgae, harvesting and processing of biomass. In the idealized case, the conversion plants are located in or near the biomass growth areas to minimize the cost of transporting biomass to the plants, of which all the non fuel effluents are recycled to the growth areas as demonstrated in Figure 1 [17].

The growth can be implemented in a microalgal farm. It would be equivalent to an isolated system with inputs of sunlight, air, CO_2_, and water. Nutrients are replenished based on their status in the growth media and the environmental controls and waste disposal problems are minimized.

Approximately, half of the dry weight of microalgal biomass is carbon [21], which is typically derived from CO_2_. Thus, producing 1 kg of algal biomass fixes 1.6 – 1.8 kg of CO_2_. The CO_2_ must be fed continuously during daylight hours. Thus, an algae farm can be located adjacent to a power plant for utilizing CO_2_ from the combustion process [17, 19, 22]. In such a circumstance, management of other gaseous emissions and wastewater by algal culture is also possible. It is because algae can remove effectively nitrogen, phosphorus, and heavy metals such as As, Cd, and Cr from aqueous solutions [19, 23]. Since emission control and wastewater management are costly and technically demanding, the use of wastewater as a source of nutrients for algae production, coupled with wastewater treatment are added environmental and economic benefits. The algal biomass produced needs to be further processed to recover the biomass. A problem associated with algal biomass is the relatively high water content. It normally requires pre-treatments to reduce the water content and increase the energy density. This requirement consequently increases the energy cost. However, direct hydrothermal liquefaction in sub-critical water conditions can be employed to convert the wet biomass to liquid fuel without reducing the water content. Overall, by adopting integration approaches, such as wastewater treatment, nutrients and heavy metals recovery by algae culture, whereby additional economic benefits are created the obstacle of high cost of biodiesel production from algae may be overcome.

## 3. Production of Microalgal Biomass

The production of microalgal biodiesel requires large quantities of algal biomass. Most of algal species are obligate phototrophs and thus require light for their growth. Several cultivation technologies that are used for production microalgal biomass have been developed by researchers and commercial producers. The phototropic microalgae are most commonly grown in open ponds and photobioreactors [18]. The open pond cultures are economically more favorable, but raise the issues of land use cost, water availability, and appropriate climatic conditions. Further, there is the problem of contamination by fungi, bacteria and protozoa and competition by other microalgae. Photobioreactors offer a closed culture environment, which is protected from direct fallout, relatively safe from invading microorganisms, where temperatures are controlled with an enhanced CO_2_ fixation that is bubbled through culture medium. This technology is relatively expensive compared to the open ponds because of the infrastructure costs. An ideal biomass production system should use the freely available sunlight. It is reported the best annual averaged productivity of open ponds was about 24 g^−1^ dry weight m^−2^ d^−1^ [24]. A productivity of 100 g^−1^ dry weight m^−2^ d^−1^ was achieved in simple 300 l culture systems [18]. This level has been viewed as deriving from the light saturation effect. The light requirement coupled with high extinction coefficient of chlorophyll in algae has necessitated the design and development of novel system for large scale growth. Experiments have also elucidated that algal biomass production can be boosted by the flashing light effect [25–26], namely by better matching photon input rate to the limiting steps of photosynthesis. Indeed, the best annual averaged productivity has been achieved in closed bioreactors. Tridici [27] has reviewed mass production in photobioreactors. Many different designs of photobioreactor have been developed, but a tubular photobioreactor seems to be most satisfactory for producing algal biomass on the scale needed for biofuel production. Closed, controlled, indoor algal photobioreactors driven by artificial light are already economical for special high-value products such as pharmaceuticals, which can be combined with production of biodiesel to reduce the cost.

## 4. Direct Liquefaction of Algae for Biodiesel Production

The microalgal biomass has relatively high water content (80–90%) and this is major bottleneck for usage in energy supply. As most other virgin biomass, the high water content and inferior heat content makes the microalgal biomass difficult to be used for heat and power generation. Thus necessitating pre-treatments to reduce water content and increase the energy density. As consequence the energy cost increases and makes the alternative less economically attractive [17, 29–31].

Direct hydrothermal liquefaction in sub-critical water conditions is a technology that can be employed to convert wet biomass material to liquid fuel. This technology is believed to mimic the natural geological processes thought to be involved in the formation of fossil fuel, but in the time scale of hours or even minutes. A number of technical terminologies have been used in the literature to refer to this technology, but it essentially utilize the high activity of water in sub-critical conditions in order to decompose biomass materials down to shorter and smaller molecular materials with a higher energy density or more valuable chemicals. Goudriaan *et al*. [32] claim the thermal efficiency (defined as the ratio of heating values of bio-crude products and feedstock plus external heat input) for the hydrothermal upgrading process (HTU^®^) of biomass of a 10 kg dry weight h^−1^ pilot plant is as high as 75%. The main product of the process is bio-crude accounting for 45% wt. of the feedstock on dry ash free basis, with a lower heating value of 30–35 MJ kg^−1^, which is compatible with fossil diesel and can be upgraded further [32]. As moist biomass can be easily heated by microwave power, a process similar to the HTU^®^ process using a novel microwave high-pressure (MHP) reactor has been developed in order to further minimize the energy consumption of the process [33].

In addition, integrated utilization of high temperature and high pressure conditions in the process of hydrothermal liquefaction of wet biomass would significantly improve the overall thermal efficiency of the process. Suitable systems for such utilization are an internal heat exchanger network, or a combined heat and power (CHP) plant. A thermodynamic study, for example, has been performed to calculate the energy efficiency of the HTU process on the basis an integrated heat exchanger network [34].

Past research in the use of hydrothermal technology for direct liquefaction of biomass was very active. Only a few of them, however, used algal biomass as feedstock for the technology. Minowa *et al*. [35] report an oil yield of about 37% (organic basis) by direct hydrothermal liquefaction at around 300°C and 10 MPa from *Dunaliella tertiolecta* with a moisture content of 78.4 wt%. The oil obtained at a reaction temperature of 340°C and holding time of 60 min had a viscosity of 150–330 mPas and a calorific value of 36 kJ g^−1^, comparable to those of fuel oil. The liquefaction technique was concluded to be a net energy producer from the energy balance. In a similar study on oil recovery from *Botryococcus braunii*, a maximum yield 64% dry wt. basis of oil was obtained by liquefaction at 300°C catalyzed by sodium carbonate [23]. Also, Aresta *et al*. [36–37] have compared different conversion techniques *viz.*, supercritical CO_2_, organic solvent extraction, pyrolysis, and hydrothermal technology for production of microalgal biodiesel. The hydrothermal liquefaction technique was more effective for extraction of microalgal biodiesel than using the supercritical carbon dioxide [36]. From these two studies, it is reasonable to believe that, among the selected techniques, the hydrothermal liquefaction is the most effective technological option for production of bio-diesel from algae. Nevertheless, due to the level of limited information in the hydrothermal liquefaction of algae, more research in this area would be needed.

## 5. Conclusions

Biofuels produced from renewable biomass are the sustainable energy resource with greatest potential for CO_2_ neutral production. The first generation biofuels produced from corn, sugarcane, and soya perform poorly in many environmentally context [38]. Innovative studies like that of Zah *et al*. [39] provide a detailed assessment of the environmental costs and benefits of different transport biofuels. Though, they did not include the second generation biofuels because of insufficient data. More studies on these lines are necessary for evaluating different biofuels. A sustainable and profitable biodiesel production from microalgae is possible. This second generation biofuel can overcome the energy and environmental needs by integrating the technologies. Large quantities of algal biomass needed for the production of biodiesel could be grown in photobioreactors combined with photonics and biotechnologies. However, more precise economic assessments of production are necessary to establish with petroleum derived fuels. The direct hydrothermal liquefaction is energy efficient technique for producing biodiesel from algae without the need to reduce the water content of the algal biomass. More research in this area is needed. The overall approach would adopt an integrated biomass-production conversion system. It includes a microalgal production at thermal power plants for sequestering CO_2_, wastewater treatment and emission control, integration of an internal heat exchanger network and utilization of high pressure and high temperatures from the conversion reactor for power generation. The residual biomass from oil extraction could be used partly as high protein animal feed and, possibly, as source of small amounts of other high-value microalgal products. Another exciting reason about microalgal oil is the potential of chemically converting it into kerosene, the basic component of jet fuel. Jet fuel now accounts for about 8% of the petroleum use with very few renewable alternatives. Ethanol is not dense enough with only half the energy per volume of the jet fuel. Biodiesel has about 80% the energy density of kerosene but can solidify at the low temperatures of high altitude flight. Thus, microalgal biofuels are a sustainable energy resource, the challenge will be the economics of production. A few companies are already underway to achieve commercial-scale production of microalgal biofuels.
